# MRSA screening in emergency department detects a minority of MRSA carriers

**DOI:** 10.1186/1757-7241-23-S1-A30

**Published:** 2015-07-16

**Authors:** Christian B Mogensen, Poul Kjældgaard, Lilli Ø Skov, Charlotte Jensen, Ming Chen

**Affiliations:** 1 Emergency Department, Southern Jutland Hospital, Aabenraa, Denmark; 2 Department of Clinical Microbiology, Southern Jutland Hospital, Aabenraa, Denmark

## Background

Methicillin-resistant *Staphylococcus aureus *(MRSA) is an emerging problem in Denmark. The National Board of Health (NBH) has issued a screening tool to identify risk situations for admitted patients, consisting of three parts: questions concerning general risk situations, special risk situations, and individual risk factors. All patients should answer the general risk situation questions, the special risk, and risk factor on indication and be tested for MRSA if a risk situation is identified. Since the majority of all acute patients are admitted to the emergency departments (ED), the ED plays a key role in prevention of in-hospital spreading of MRSA. The aim of this study was to estimate the prevalence of MRSA among all acutely admitted ED patients and to evaluate the ability of the NBH screening tool to detect MRSA patients.

## Methods

All patients more than 10 years, acutely admitted to the ED in the Hospital of Southern Jutland during a three months period were requested to answer all the NBH questions and a nasal and pharyngeal swab was obtained for MRSA culture.

## Results

1,945 patients above 10 years were admitted, 1,660 patients (85%) were asked to participate and 1,220 accepted (63% of all admissions). 11 patients had MRSA (0.9%). The performance of the general risk situation screening to detect MRSA carriers was: sensitivity 18% (95% CI: 2-52%), specificity 96% (94-97%), positive predictive value (PPV) 4% (0.4-12%) and negative predictive value (NPV) 99% (99-99.6%), likelihood ratio for positive test (LHR+) 4.0 (1-14) and likelihood ratio for negative test LHR(-) 0.9 (0.7-1.1). For the combined general, specific, and individual risk factors the sensitivity was 46% (17-77%), specificity 60% (57-63%) PPV 1% (0.3-2.4%) NPV 99% ( 98-99.7%), LHR (+) 1.1 (0.6-2) and LHR (-) 0.9 (0.5-1.6).

## Conclusion

In this ED 0.9% of the patients had MRSA. Less than every fifth will be detected by the general screening questions and less than half if all general, specific, and individual questions are used. We conclude that the majority of MRSA carriers acutely admitted to the ED will remain undetected.

